# Mapping the helix arrangement of the reconstituted ETR1 ethylene receptor transmembrane domain by EPR spectroscopy†

**DOI:** 10.1039/d2ra00604a

**Published:** 2022-03-04

**Authors:** Anandi Kugele, Buket Uzun, Lena Müller, Stephan Schott-Verdugo, Holger Gohlke, Georg Groth, Malte Drescher

**Affiliations:** Department of Chemistry and Konstanz Research School Chemical Biology (KoRS-CB), University of Konstanz Universitätsstraße 10 78457 Konstanz Germany malte.drescher@uni-konstanz.de; Institute of Biochemical Plant Physiology, Heinrich Heine University Düsseldorf Universitätsstraße 1 40225 Düsseldorf Germany georg.groth@hhu.de; John von Neumann Institute for Computing (NIC), Jülich Supercomputing Centre (JSC), Institute of Biological Information Processing (IBI-7: Structural Biochemistry), Institute of Bio- and Geosciences (IBG-4: Bioinformatics), Forschungszentrum Jülich GmbH 52425 Jülich Germany; Institute for Pharmaceutical and Medicinal Chemistry, Heinrich Heine University Düsseldorf 40225 Düsseldorf Germany

## Abstract

The plant ethylene receptor ETR1 is a key player in the perception of the phytohormone and subsequent downstream ethylene signal transmission, crucial for processes such as ripening, senescence and abscission. However, to date, there is sparse structural knowledge about the transmembrane sensor domain (TMD) of ETR1 that is responsible for the binding of the plant hormone and initiates the downstream signal transmission. Sequence information and *ab initio* modelling suggest that the TMD consists of three transmembrane helices. Here, we combined site-directed spin labelling with electron paramagnetic resonance spectroscopy and obtained distance restraints for liposome-reconstituted ETR1_TMD on the orientation and arrangement of the transmembrane helices. We used these data to scrutinize different computational structure predictions of the TMD.

## Abstract 2

The ethylene receptor 1 transmembrane domain was site-directedly spin labelled to obtain distance restraints.

## Graphical abstract

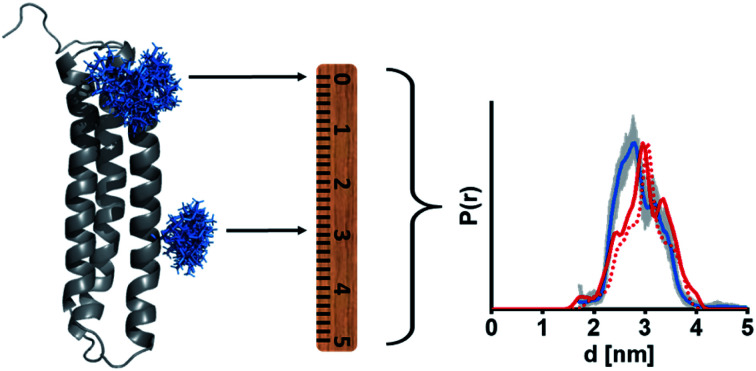

## Introduction

**Fig. 1:**
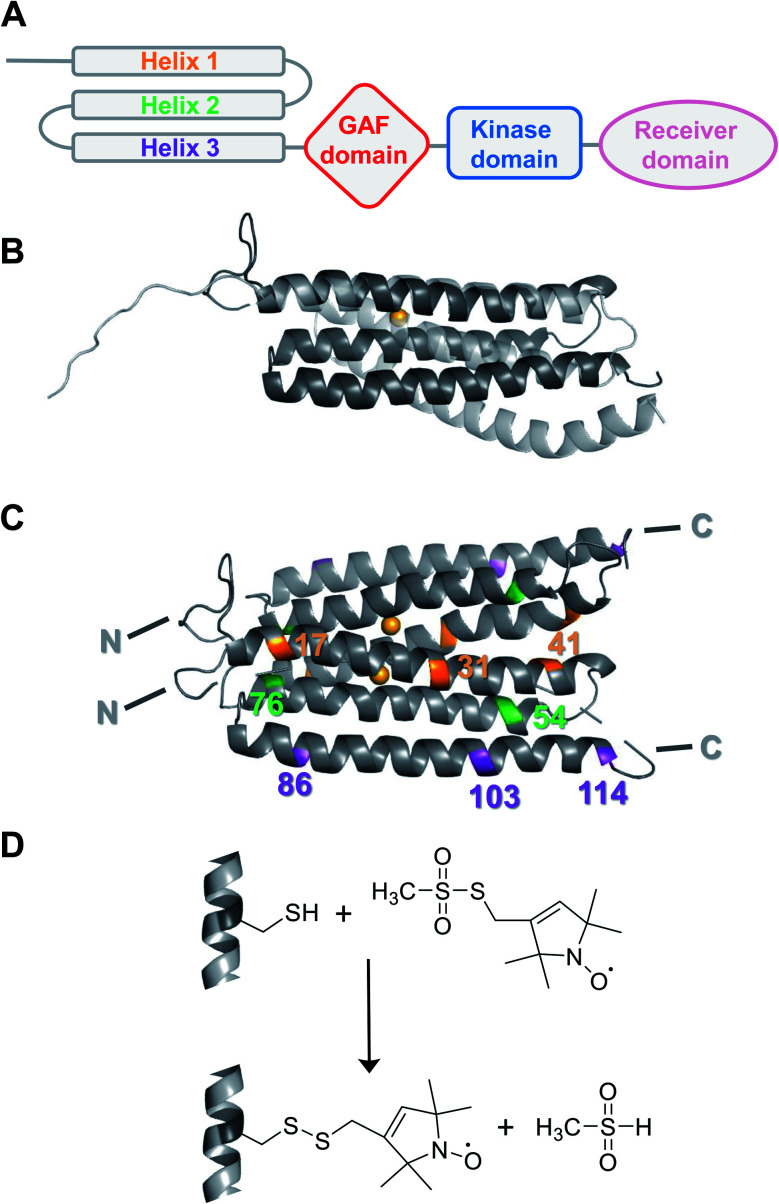
(A) Schematic organization of the modular structure of an ETR1 monomer. (B) Overlaid structural models of ETR1_TMD as predicted *ab initio* by Schott-Verdugo *et al.* (2019, dark grey) and AlphaFold (2021, light grey). The copper(i) ion included in the *ab initio* model is depicted as light orange sphere. (C) Structural model of the ETR1_TMD dimer as predicted by Schott-Verdugo *et al.* (2019). Residues used in this study for cysteine mutagenesis and spin labelling were highlighted, and the copper(i) ions are depicted as light orange spheres. (D) Spin labelling of cysteines using the methanethiosulfonate spin label (MTSSL) forms the side chain R1.

Plant hormones (phytohormones) are the key players in integrating developmental signals and responses to the environment. In particular, the gaseous ethylene stimulates several physiological processes such as growth, senescence, pathogen responses, and fruit ripening, and is perceived by the ethylene receptor family.^1^ Ethylene sensing leads to suppression of a downstream signalling cascade and subsequent activation of gene expression responsible for ethylene-induced biological responses.^2,3^ In *Arabidopsis thaliana* (*A. thaliana*) five ethylene receptor isoforms (ETR1, ERS1, ETR2, ERS2, EIN4) have been identified.^4^ They share a conserved modular structure, consisting of an N-terminal ethylene binding transmembrane sensor domain (TMD), a GAF (cGMP-specific phosphodiesterases, adenylyl cyclase and FhlA), and a catalytic transmitter domain;^4–7^ ETR1, ETR2 and EIN4 additionally possess a receiver domain at the C-terminus (Fig. 1A).^8^ All ethylene receptors are involved in ethylene signalling with partially overlapping roles.^9^ However, more detailed structural and mechanistic knowledge is required to answer open questions including receptor output and downstream signalling, which are still largely unknown.

While a structural model of the complete cytosolic domains of receptors ERS1 and ETR1 has been obtained by crystal structure analysis and low-resolution SAXS,^10,11^ the structure of the TMD has not been resolved experimentally yet. Notably, the first *ab initio* structural model of the ETR1_TMD was recently predicted and refined by tryptophan substitution scanning mutagenesis (Fig. 1B and C).^12^ It is generally agreed, that the hydrophobic TMD monomer is composed of three membrane-spanning α-helices,^12,13^ that, besides ethylene sensing,^14^ also serve for localization of the ethylene receptor at the endoplasmic reticulum,^15^ and for generation of higher-order complexes.^16,17^

In this study, Electron Paramagnetic Resonance (EPR) spectroscopy was introduced as a tool to investigate ethylene receptors, in particular the ETR1_TMD (residues 1–157). EPR spectroscopy in combination with site-directed spin labelling (SDSL) is a valuable tool to monitor protein structure and dynamics in a background-free mode,^18^ and has also been applied to membrane proteins.^19–22^ We employed a particular EPR technique, namely double electron–electron resonance (DEER) spectroscopy,^23^ to determine distance restraints between strategically positioned spin labels in the reconstituted ETR1_TMD. We used these distance restraints to scrutinize currently available computational models of the ETR1_TMD – the dimeric *ab initio* structural model by Schott-Verdugo *et al.*^12^ and the artificial intelligence-derived AlphaFold^24^ structural model (UniProt P49333, Fig. 1B).

## Results and discussion

To obtain suitable ETR1_TMD constructs for thiol-mediated spin labelling, native cysteines were replaced in the *A. thaliana* ETR1 receptor by serine residues (ETR1_TMD_C4S/C6S/C65S/C99S, referred to as ETR1_ΔC in the following). Mutation of the native cysteines did not perturb the structure of ETR1_TMD (Fig. S12, ESI†). New cysteines for SDSL were installed at strategically positioned sites (Fig. 1C, 3C and S1, ESI†). Based on the knowledge about the membrane-embedded α-helix bundle,^12,13^ one single and nine double cysteine-mutants were designed within the three-helix bundle of a monomer (Fig. S1 and S3,† ESI). They allow for distance determinations either within an individual helix (intrahelical) or between the three helices in the ETR1_TMD monomer (interhelical).

After expression in *Escherichia coli* and purification of ETR1_TMD,^12^ disulfide bridges were reduced by dithiothreitol (DTT) to enhance subsequent labelling efficiency. Site-directed spin-labelling (SDSL) of cysteine residues with the methanethiosulfonate spin label (MTSSL) was performed in the presence of the detergent *n*-hexadecyl-phosphocholine, resulting in the spin-labelled side chain R1 (Fig. 1D; for detailed procedures see ESI†).^22,25^ After removal of excess label, continuous-wave (cw)-EPR spectra of the mutants were recorded. Analysis of spin concentrations revealed no background labelling of ETR1_ΔC and virtually quantitative labelling of the cysteine variants, indicating that the selected residues are well accessible for spin labelling (Fig. S3, ESI†).

**Fig. 2:**
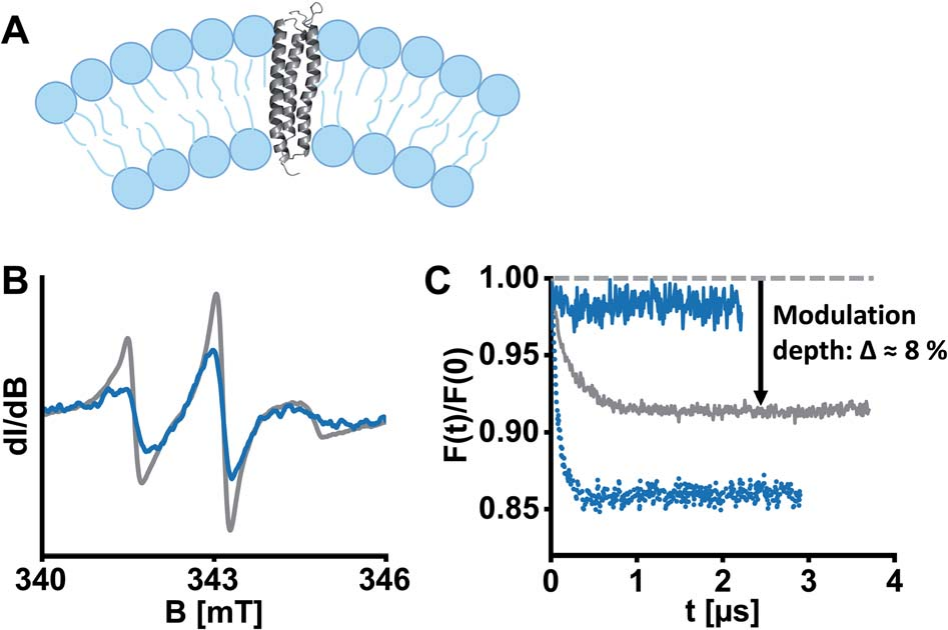
ETR1 variants were reconstituted into DMPC large unilamellar vesicles for EPR experiments. (A) Schematic liposome cross section with incorporated ETR1. Possible dimer/oligomer formation was omitted in this representation for the sake of clarity. (B) Cw-EPR spectra of ETR1_L17C→R1 before (grey) and after (blue) reconstitution, normalized to the area under the curve. (C) DEER form factors after background subtraction for ETR1_L17C→R1 in detergent-containing buffer (grey, *Δ* ≈ 8%), after reconstitution (dotted blue, *Δ* ≈ 14%), and after diamagnetic dilution in combination with reconstitution (solid blue, *Δ* ≈ 2%). Corresponding DEER raw data is shown in Fig. S11, ESI.†

ETR1 in its functional form occurs as homo-^16^ or heterodimer^26^ or as part of larger protein complexes,^27^ mediated by disulfide linkage^16^ or in a cysteine-independent manner.^17^ To assess the potentially dimeric state of our spin-labelled ETR1 constructs, we shock-froze a sample of singly spin-labelled ETR1_ΔC_L17C→R1 in the detergent-containing buffer supplemented with 20% deuterated glycerol. To detect potential intermolecular interactions, we performed a four-pulse DEER measurement, and the form factor exhibited a modulation depth deviant from zero (*Δ* ≈ 8%, Fig. 2C, grey curve). The modulation depth is an indicator for dipolar interaction. This indicates moderate cysteine-independent interactions between neighboring ETR1 monomers or partial aggregation, even though mainly monomeric ETR1 was confirmed by non-reducing SDS-PAGE (Fig. S4, ESI†). For doubly spin-labelled variants, these interactions would complicate accurate evaluation of distances.

To exclude aggregation and ensure functional and structural integrity, a reconstitution approach in liposomes was pursued (Fig. 2A). Liposomes closely resemble cellular membranes, and have been applied to study membrane proteins by EPR spectroscopy several times.^28^ To this aim, we prepared homogenously 100 nm-sized large unilamellar vesicles (LUVs) composed of the phospholipid 1,2-dimyristoyl-*sn*-glycero-3-phosphocholine (DMPC) by extrusion, and then partially solubilized the preformed LUVs with Triton X-100.^29^ Afterwards, ETR1_ΔC_L17C→R1 was added to the LUVs in a standard molar lipid-to-protein ratio of 2000. Following stepwise removal of detergents with polystyrene beads, we collected the proteoliposomes by ultracentrifugation. Complete incorporation of ETR1 was confirmed by cw-EPR spectroscopy of the proteoliposome pellet and the supernatant (Fig. S3, ESI†). The spectra further revealed distinct broadening upon incorporation into vesicles, reflecting restricted motion of ETR1 and of the attached spin label (Fig. 2B).

To investigate the effect of reconstitution, DEER of the singly labelled variant ETR1_ΔC_L17C→R1 was measured. Interestingly, the modulation depth had even increased to *Δ* ≈ 14% (Fig. 2C, dotted blue curve), indicating intermolecular interactions. This implies that multimolecular species are also present in the proteoliposomes, complicating the evaluation of DEER measurements. Only when spin-labelled proteins were diamagnetically diluted with ETR1_ΔC prior to reconstitution (in a ratio of one labelled variant plus five ETR1_ΔC), the modulation depth was reduced to *Δ* ≈ 2% (Fig. 2C, solid blue curve). Under these conditions, multimolecular species such as oligomers consisting of ETR1_ΔC_L17C→R1 and ETR1_ΔC are still formed. The combination of diamagnetic dilution and reconstitution, however, sufficiently prevents detecting intermolecular spin–spin interactions and allows resolving only intramolecular distances. Notably, in this approach a superposition of distance distributions from monomers and dimers is measured, and the conformation of a monomer within a dimer may be different then the conformation of the monomeric ETR1.

In the next step, DEER traces were recorded for doubly labelled ETR1 diamagnetically diluted in proteoliposomes.

**Fig. 3:**
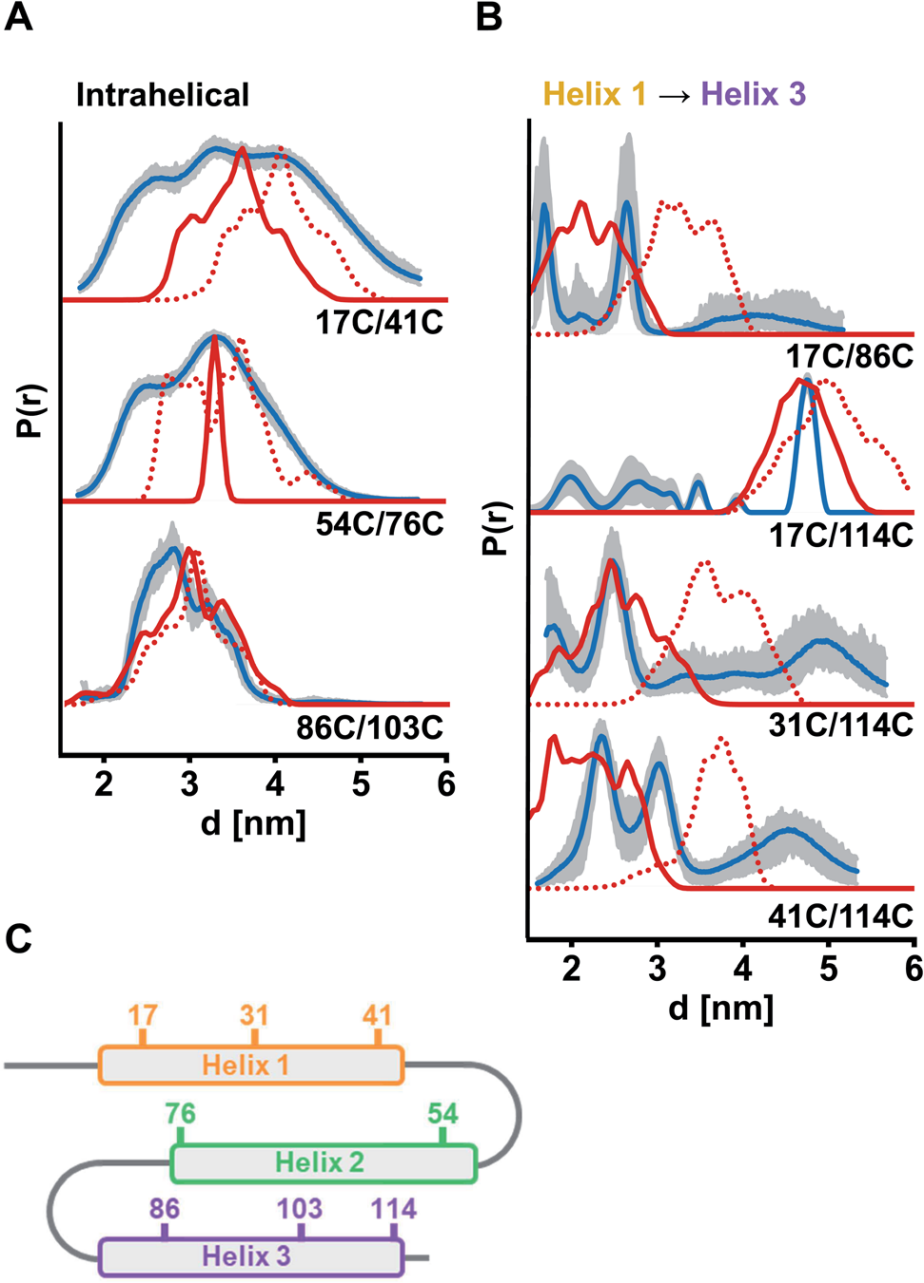
Experimental distance distributions in the ETR1_TMD obtained by DEER measurements (blue) with validation (grey area). Simulated distance distributions by MMM based on the model by Schott-Verdugo *et al.* (2019, red) and by AlphaFold (2021, red dotted) are indicated. Distance distributions were scaled to their maximum. (A) Intrahelical distances. (B) Interhelical distances between helix 1 and helix 3. (C) Schematic representation of the ETR1_TMD and spin-labelled sites used for DEER distance determinations. DEER raw data and interhelical distances between helix 1 and helix 2 are given in Fig. S6, ESI.†

For verification of helix integrity, at first distance distributions between two labelled sites per helix were measured (ETR1_ΔC_L17C/Y41C→R1, ETR1_ΔC_V54C/F76C→R1 and ETR1_ΔC_V86C/L103C→R1). The modulation depths were in the expected range (*Δ* ≈ 39–46%, Fig. S6A, ESI†). To provide a reference for assessing experimentally derived distance distributions, *in silico* simulations based on current structural models of ETR1 (ref. 12 and 24) were generated using the Multiscale Modeling of Macromolecules^30^ software (MMM). *In silico* labelling of sites 54 and 76 was sterically hindered (Fig. S9B, ESI†), which resulted in an artificially narrowed simulation (Fig. 3A); that was, however, not the case to such extent when taking the AlphaFold model^24^ as basis (Fig. 3A and S9B, ESI†). Altogether, for the three intrahelical distance restraints the shape of the distributions and the mean distance conform well to the expectation, although broader in shape than expected for inherently rigid α-helices (Fig. 3A). Especially for helix 1 this suggests, that it is not as structured as expected and as proposed by the models.

Moreover, interhelical distances were determined to address the topological arrangement and the orientation of the α-helices towards each other. However, the data quality for distance distributions measured between helices 1 and 2 was too low to reliably analyse the extracted distances (ETR1_ΔC_L17C/V54C→R1 and ETR1_ΔC_A31C/F76C→R1; Fig. S6B, ESI†). However, the raw data do indicate long and broad distance distributions instead of the expected short distances. Moreover, the modulation depth of these measurements was reduced to *Δ* ≈ 12% (Fig. S6B, ESI†), which may originate from distances too long or too short (approx. <1.8 or >5.2 nm under our experimental conditions) to be resolved by the acquired DEER traces. By cw measurements at 120 K (ref. 31) such short distances were excluded for ETR1_ΔC_L17C/V54C→R1 (Fig. S10†). Hence, the observed DEER data could be the result of displacement of helix 2 with respect to Schott-Verdugo *et al.*^12^ enabled by the flexible loops (schematically represented in Fig. 3C). The AlphaFold model also supports such a displacement.

The most significant differences between the two model structures are visible in the localization of helix 3 with respect to helix 1 (Fig. 1B). To assess this experimentally, we focused on measurements between helices 1 and 3 (ETR1_ΔC_L17C/V86C→R1, ETR1_ΔC_L17C/S114C→R1, ETR1_ΔC_A31C/S114C→R1 and ETR1_ΔC_Y41C/S114C→R1). In this series of data, especially for ETR1_ΔC_L17C/S114C→R1, the width of the distance distribution cannot be reliably extracted due to short length of the dipolar evolution time,^32^ which is typical for DEER measurements in membranes.^28^ However, regarding the main distance, measurements between helices 1 and 3 are in good agreement with the *ab initio* model (Fig. 3B). The AlphaFold model exhibits a looser and less parallel arrangement of the helices (Fig. 1B and S9A, ESI†), and consequently tends to longer distances (Fig. 3B).

**Fig. 4:**
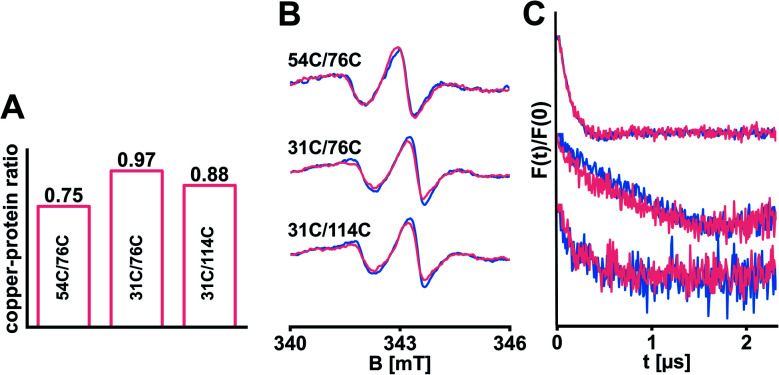
Selected ETR1 variants were loaded with copper(i). (A) The copper-to-protein ratio was determined spectrophotometrically. (B) Cw-EPR spectra recorded after diamagnetic dilution and reconstitution of samples with (pink) and without (blue) copper(i). (C) Corresponding DEER-derived form factors after background subtraction, scaled to the modulation depth. DEER raw data is shown in Fig. S8, ESI.†

Copper(i) is an essential cofactor to mediate high-affinity ethylene binding^33,34^ and was included in the *in silico* model,^12^ but absent in our experiments so far. In the field of ethylene receptors there are still open questions regarding transfer routes and coordination of copper(i).^1,35^ We reasoned that bound copper(i) might have a stabilizing impact on the structure of ETR1. Consequently, we transferred copper(i) from the bicinchoninic acid (BCA)-based BCA_2_-Cu(i) complex (Fig. S7A, ESI†) to selected ETR1 variants as described by Schott-Verdugo *et al.*^12^ (with slight changes to protect the spin labels, see ESI†), before removing excess copper(i). To determine the copper-to-protein stoichiometry, ETR1_TMD was denatured to release the copper(i). These ions were trapped in the BCA_2_–Cu(i) complex and quantified spectrophotometrically by measuring the absorption of the complex at 562 nm.^12^ In analogy to previous findings,^12,36^ our experiment revealed effective loading of approx. 0.75–0.97 copper(i) per ETR1 monomer (Fig. 4A and S7, ESI†). After reconstituting the copper-loaded variants, cw-EPR spectra (Fig. 4B) as well as the obtained DEER data (Fig. 4C) overlaid with the data acquired in absence of copper(i). This means, that no global changes in the ETR1 structure upon copper(i) loading were observed.

## Conclusions

In summary, our data give rise to four key findings regarding the structure of the ETR1_TMD. First, the experimental distance restraints are altogether in better agreement with the *ab initio* structural model^12^ than with the AlphaFold^24^ prediction. Second, intrahelical flexibility is higher than expected from rigid models under the condition of sample preparation. Third, our data suggest displacement of helix 2 towards the C-terminal direction enabled by loop flexibility. Fourth, loading of the ETR1_TMD with copper(i) does not induce significant conformational changes.

To summarize, we have established an SDSL-EPR approach to study the TMD of ETR1, constituting a complementary tool to evaluate its structural conformation. This study reveals large potential to investigate ETR1 in the presence of its various interacting partners and represents a valuable building block towards an in-depth mechanistic understanding of ethylene signalling in plants.

## Conflicts of interest

There are no conflicts to declare.

## Supplementary Material

RA-012-D2RA00604A-s001
