# Supplementary material for: Mapping the helix arrangement of the reconstituted ETR1 ethylene receptor transmembrane domain by EPR spectroscopy
Source: RSC Adv. 2022 Mar 4;12(12):7352–6. doi: 10.1039/d2ra00604a (PMC8982231; doi:10.1039/d2ra00604a)
Supplement: RA-012-D2RA00604A-s001 [file RA-012-D2RA00604A-s001.pdf]

Electronic Supplementary Material (ESI) for RSC Advances.
This journal is © The Royal Society of Chemistry 2022

Supporting Information for "Mapping the helix arrangement of the reconstituted ETR1 ethylene receptor transmembrane domain by EPR spectroscopy"

Anandi Kugele,‡ Buket Uzun,‡ Lena Müller, Stephan Schott-Verdugo, Holger Gohlke, Georg Groth* and Malte Drescher*

Table of Contents

Material and Methods **2**

Expression and purification 2

Spin labelling 2

Preparation of Large Unilamellar Vesicles (LUVs) 3

Reconstitution 3

Dynamic Light Scattering (DLS) 3

Continuous wave (cw)-EPR 4

Double Electron-Electron Resonance (DEER) 4

Copper(I) loading 5

Circular Dichroism (CD) Spectroscopy 5

Supplementary Figures **6**

Figure S1: Primary sequence of the ETR1_TMD 6

Figure S2: Oligonucleotides used for site-directed mutagenesis 7

Figure S3: Cw-EPR spectra before and after reconstitution 8

Figure S4: Non-reducing PAGE 9

Figure S5: DLS data of LUVs and proteoliposomes 10

Figure S6: DEER data of double mutants 11

Figure S7: Determination of copper-protein-stoichiometry 12

Figure S8: DEER data of copper(I)-loaded variants 13

Figure S9: Structural models used for distance simulations 14

Figure S10: Low-temperature cw-EPR spectra 15

Figure S11: DEER data of single mutant 15

Figure S12: CD spectra 16

Supplementary References **17**

Material and Methods

Expression and purification

Cloning of ETR1_TMD mutants, expression in *E. coli* and purification were performed as described before.[1] All oligonucleotides used for cloning are given in Figure S2.

Spin labelling

A 100 mM stock solution of MTSSL (*S*-[1-oxyl-2,2,5,5-tetramethyl-2,5-dihydro-1*H*-pyrrol-3-yl] methyl methanesulfonothioate; SIGMA) in DMSO (SIGMA) was prepared, aliquoted and stored at -80 °C. A 1 M DTT (SIGMA) stock solution was prepared in MilliQ-water, aliquoted and stored at -20 °C.
ETR1 mutants were diluted with ETR1 buffer (50 mM TRIS (SIGMA), 200 mM NaCl (Fisher Scientific), pH 8) to 30 µM, and 1 vol-% DTT stock solution was added to reduce cysteine bridges. Samples were incubated at 25 °C and 500 rpm for 1 h in an Eppendorf ThermoMixer. DTT was removed in spin filters (Amicon Ultra-0.5 mL Centrifugal Filters, 3K cutoff, Merck) by washing 12 times with ETR1 buffer at 14.000 g and 10 °C. An appropriate volume of ETR1 buffer supplemented with 0.015 % hexadecylphosphocholine (Fos-choline 16, Glycon) was added to yield 30 µM of ETR1 for spin labelling. Samples were spin labelled with a 40-fold molar excess of MTSSL[2] overnight at 4 °C and 600 rpm. Residual MTSSL was removed by washing 12 times with ETR1 buffer in spin filters as described above. After sample collection, room-temperature cw-EPR spectra were recorded to ensure complete labelling and sufficient removal of excess MTSSL. ETR1 concentration was determined photometrically with the use of an Eppendorf BioPhotometer D30 via absorption at 280 nm ($A_{280/1mm}$) using the extinction coefficients given in the table below (using ETR1 buffer as blank). Extinction coefficients were determined with the Expasy tool ProtParam.[3] Samples containing 20 vol-% glycerol (SIGMA) as cryoprotectant were frozen at -80 °C until reconstitution.

**Mutant**	**Molar extinction coefficient ε [M^{-1} cm^{-1}]**	**Molecular weight MW [Da]**
**ETR1_ΔC**	23950	20483.81
**ETR1_ΔC_L17C**	23950	20473.79
**ETR1_ΔC_V54C/F76C**	24075	20443.78
**ETR1_ΔC_L17C/V54C**	24075	20477.8
**ETR1_ΔC_L17C/V86C**	24075	20477.8
**ETR1_ΔC_L17C/S114C**	24075	20489.85
**ETR1_ΔC_L17C/Y41C**	22585	20413.75
**ETR1_ΔC_V86C/L103C**	24075	20477.8
**ETR1_ΔC_Y41C/S114C**	22585	20439.84
**ETR1_ΔC_A31C/F76C**	24075	20471.83
**ETR1_ΔC_A31C/S114C**	24075	20531.93

Preparation of Large Unilamellar Vesicles (LUVs)

For one DEER sample, 15 mg (22.14 µmol) of DMPC (1,2-dimyristoyl-*sn*-glycero-3-phosphocholine, Avanti Polar Lipids) with a molar mass of M = 677.5 g/mol were dissolved in 1.5 mL chloroform (spectroscopic grade, SIGMA) in a glass tube. While rotating by hand, the chloroform was removed under a nitrogen stream, and the resulting lipid layer was evaporated in vacuum overnight. The lipids were rehydrated in 3 mL ETR1 buffer to yield a concentration of 5 mg/mL, sonicated, and vortexed for 10 min, before incubation in the dark for 45 min at room temperature. Additionally, five freeze-thaw cycles were performed to form large unilamellar vesicles (LUVs). 100 nm-sized LUVs were then prepared by 19-fold extrusion through a layer of 100 nm polycarbonate film (Whatman) in a handheld extruder (Avanti, Polar Lipids). DLS was measured as described below to confirm size and homogeneity (DLS data is shown in Figure S5). Vesicles were stored for usually one day in the fridge until used for reconstitution.

Reconstitution

To prepare diamagnetically diluted samples, spin-labelled mutants were mixed with ETR1_ΔC in a molar ratio of 1:5. This corresponds to 1.83 nmol spin-labelled ETR1 plus 9.15 nmol ETR1_ΔC.
For one protein sample (sufficient for one cw-EPR and one DEER measurement), one portion of LUVs was prepared as described above (from 15 mg DMPC in 3 mL volume). LUVs were mixed with 191 µL of 10 % Triton X-100 (SIGMA) solution (predissolved in ETR1 buffer) for partial solubilization of the LUVs,[4] and incubated for 1 h at 22 °C without shaking. The LUVs were then merged with the ETR1 mixture in a standard lipid-to-protein ratio of 80:1 (w/w; corresponding to a molar lipid-to-protein ratio of approx. 2000), and divided into two 2 mL microcentrifuge tubes. The mixture was incubated for 1 h at 22 °C without shaking in the dark. For detergent removal, the adsorption technique using Bio-Beads SM-2 was pursued. For this purpose, 0.5 g of Bio-Beads SM-2 (BIO-RAD) per microcentrifuge tube (1 g per sample) were washed three times with methanol (SIGMA, spectroscopic grade), followed by five times washing with MQ-H_2O. The beads were stored in water and used the same day. The beads were added to the samples in four portions with a small spoon, followed by vigorous shaking for 1 h, 2 h, 2 h, and overnight. The samples became turbid during this process, as proteoliposomes are formed. The next day, the Bio-Beads SM-2 were removed in empty 0.8 mL Pierce centrifuge columns (Thermo Fisher Scientific), and the supernatant was collected in 1.5 mL ultracentrifugation tubes (Beckman Coulter Microfuge Tube Polypropylene). Proteoliposomes were collected by ultracentrifugation in a Beckman Coulter Optima MAX-XP ultracentrifuge for 1 h at 100.000 g and 4 °C. The pellet was resuspended in approx. 5 µL D_2O (SIGMA), and cw-EPR spectra of the pellet and the supernatant were measured to ensure that the nitroxide signal is found only in the pellet (Figure S3). 48 µL of the sample were mixed with 12 µL of deuterated glycerol (≙ 20 vol-%) and filled into 3 mm outer diameter quartz tubes. Samples were shock-frozen in liquid nitrogen and stored at - 80 °C until DEER measurement. Additionally, DLS was measured as described below to confirm size and homogeneity (DLS data is shown in Figure S5).

Dynamic Light Scattering (DLS)

Vesicle size was confirmed by Dynamic light scattering (DLS) in a Zetasizer nano ZS spectrometer, Malvern Instruments Ltd., at 298 K. For this purpose, 3 µL of LUVs or 1.5 µL of proteoliposomes,

respectively, were dispensed in 1 mL ETR1 buffer and filled into a 1 cm disposable polycarbonate cuvette. A refractive index of 1.44 was used and 10 scans each were accumulated. Data is shown in Figure S5.

Continuous wave (cw)-EPR

Cw-EPR spectra at room temperature (approx. 22 °C) were recorded at a Bruker EMXnano X-band continuous wave EPR spectrometer (with a cylindric cavity mode TM1110) without temperature regulation. Typical sample volumes of 30 µL were filled into a glass capillary (HIRSCHMANN ringcaps; inner diameter 1.02 mm). Spectra were recorded at a modulation amplitude of 1 G, microwave attenuation 15 dB corresponding to a power of 3.162 mW, and a sweep width of 200 G. 10 scans of 80 sec scan time each for spin-labelled protein and 30 scans of reconstituted samples were accumulated to improve the signal-to-noise ratio. Quantitative spin concentrations of samples were obtained with the use of the built-in EMXnano reference-free spin counting module (Xenon software, Bruker). The labelling efficiency was estimated as the ratio of spin/protein. Spectra were analyzed with MATLAB R2019b (The MatWorks, Inc. 3 Apple Hill Drive, Natick, MA 01760-2098, USA) and plotted with OriginLab 2018G (OriginLab Corporation, Northampton, MA, USA). Normalized spectra were obtained by dividing the spectra by area.
Cw-EPR spectra of selected mutants were recorded at 120 K to detect short distances < 2 nm (Figure S10).[5] Therefore, a Magnettech ESR5000 X-band spectrometer (Bruker) equipped with a TC H04 temperature controller was utilized. Measurements were acquired with 35 dB microwave attenuation, 0.35 mT modulation and 60 sec sweep time. 250 Scans each were accumulated to increase the signal-to-noise ratio. Spectra were baseline-corrected in Origin 2018.

Double Electron-Electron Resonance (DEER)

Distance measurements were performed in Q-band (34 GHz) using an Elexsys E580 spectrometer (Bruker Biospin) operating with a SpinJet AWG unit (Bruker Biospin) and a 150 W pulsed traveling-wave tube (TWT) amplifier (Applied Systems Engineering). The spectrometer is equipped with the EPR Flexline helium recirculation system (CE-FLEX-4K-0110, Bruker Biospin, ColdEdge Technologies) including a cold head (expander, SRDK-408D2) and a F-70H compressor (SHI cryogenics), controlled by an Oxford Instruments Mercury ITC. Samples were measured at 50 K.
For measurements of diamagnetically diluted proteoliposomes, 4-pulse DEER with a Gaussian observer pulse (pulse length optimized for every sample, ranging from $\pi_{observer}$ = 48-76 ns) and a hyperbolic secant HS(1,1) pump pulse[6] (π_{pump} = 100 ns) were employed. A frequency offset of minus 80 MHz (resulting in 33.92 GHz) was used for observer pulses. A shot repetition rate of 4 ms and the eight-step phase cycle [x](x)x_px as proposed by Tait and Stoll[7] were employed. DEER measurements were typically recorded for 22 h (up to 50 h for samples with low echo intensity) to improve the signal-to-noise ratio.

The data were processed using MATLAB R2018a and the DeerAnalysis2019 software[8]. Distance distributions were obtained by a one-step procedure as recommended by Schiemann et al.,[9] using artificial neural network analysis (DEERNet)[10].

Copper(I) loading

Buffer containing 50 mM TRIS, 200 mM NaCl and bicinchoninic acid (BCA, 2.5 mM; for BCA structure see Figure S7A) at pH 7.5 was degassed in an excicator under constant stirring to prevent oxidation of copper(I). Note that the addition of ascorbate as reducing agent would destroy nitroxide signals of spin-labelled proteins. Cu(I) (1.2 mM) was then quickly dissolved in this buffer, and spin-labelled ETR1_TMD mutants were saturated until deeply purple-stained. Samples were incubated for 5 min, before excess BCA_2-Cu(I) was removed in PD-10 columns. Samples were concentrated in spin filters (Amicon Ultra-0.5 mL Centrifugal Filters, 3K cutoff, Merck) and immediately used to determine copper-protein stoichiometry and for reconstitution, as described above. Samples loaded with Cu(I) should not be stored longer than 1 week at -80 °C containing 20 vol-% glycerol.
To determine copper-protein stoichiometries, samples were denatured with SDS (20 % w/v) and cooked at 95 °C for 20 min in the presence of 2 mM BCA to trap the Cu(I) released from the ETR1_TMD. Absorbance of the BCA_2-Cu(I) complex was then measured by absorption at 562 nm in a microplate reader (SPARK, Tecan). Cu(I) concentration was quantified by using a standard curve of BCA_2-Cu(I) (Figure S7).

Circular Dichroism (CD) Spectroscopy

CD spectra (Figure S12) of wild type and cysteine-free mutant of ETR1_TMD were recorded from 200 - 250 nm at room temperature using a Jasco-715 spectropolarimeter (Jasco GmbH, Gross-Umstadt, Germany). Both proteins were provided at 0.3 mg/ml concentration in a buffer consisting of 50 mM potassium phosphate buffer and 0.015 % (w/v) Fos-choline 16 pH 8.0. A total of 10 spectra were accumulated for each sample at 0.1 nm step resolution, 50 nm/min scan speed and 1 nm bandwidth. A cylindrical quartz cuvette (Hellma GmbH & Co. KG, Muellheim) with a path length of 1 mm and a volume of 200 µl was used in these measurements. Secondary structure was computed from the CD data obtained of both proteins by the BeStSel web server.[11-13]

Supplementary Figures

Figure S1: Primary sequence of the ETR1_TMD

10 20 30 40 50

MEV**C**N**C**IEPQ WPADEL**L**MKY QYISDFFIAI **A**YFSIPLELI **Y**FVKKSAVFP

60 70 80 90 100

YRW**V**LVQFGA FIVL**C**GATHL INLWT**F**TTHS RTVAL**V**MTTA KVLTAVVS**C**A

110 120 130 140 150

TA**L**MLVHIIP DLL**S**VKTREL FLKNKAAELD REMGLIRTQE ETGRHVRMLT

157

HEIRSTL

Amino acid sequence of residues 1-157 of wildtype *A. thaliana* ETR1_TMD (taken from UniProt P49333, ETR1_ARATH). Native cysteine residues marked in bold black were replaced with serine in this study to ensure background-free spin labelling. The residues marked in orange (helix 1), green (helix 2), and purple (helix 3) per mutant were (in different combinations) replaced with cysteine for EPR measurements.

Figure S2: Oligonucleotides used for site-directed mutagenesis

Primer	Sequence
**C4S_C6S_forward**	GAAGTCAGCAATAGTATTGAACCGC
**C4S_C6S_reverse**	CATATGACGACCTTCGATATGGC
**L17C_forward**	CATCTCCGATTTCTTCATTGCGATT
**L17C_reverse**	TATTGGTATTTCATACACAATTCATCCG
**A31C_forward**	TCCTCTTGAGTTGATTTACTTTGTGAA
**A31C_reverse**	ATCGAAAAATAGCAAATCGCAATGAAG
**Y41C_forward**	AATCAGCCGTGTTTCCGTATAGAT
**Y41C_reverse**	TCTTCACAAAGCAAATCAACTCAAGAG
**V54C_forward**	TCCGTATAGATGGTGTCTTGTTCAGTT
**V54C_reverse**	AACACGGCTGATTTCTTCACAAAGTAA
**C65S_forward**	TTTTATCGTTCTTTCTGGAGCAACTCATCT
**C65S_reverse**	GCACCAAACTGAACAAGTACCCATCTATAC
**F76C_forward**	AACTTATGGACTTGCACTACGCATTCG
**F76C_reverse**	GTTCTTTCTGGAGCACCTCATCTTATT
**V86C_forward**	ACCGTGGCGCTTTGTATGACTACCG
**V86C_reverse**	TCTCGAATGCGTAGTGAAAGTCCATAA
**C99S_forward**	GTTAACCGCTGTTGTCTCGTCTGCTACT
**C99S_reverse**	ACCTTCGCGGTAGTCATCACAAGC
**L103C_forward**	CGTCTGCTACTGCGTGTATGCTTGTTC
**L103C_reverse**	AGACAACAGCGGTTAACACCTTCG
**S114C_forward**	TCCTGATCTTTTGTGTGTTAAGACTCG
**S114C_reverse**	ATAATATGAACAAGCATCAACGCAGTAG

Figure S3: Cw-EPR spectra before and after reconstitution

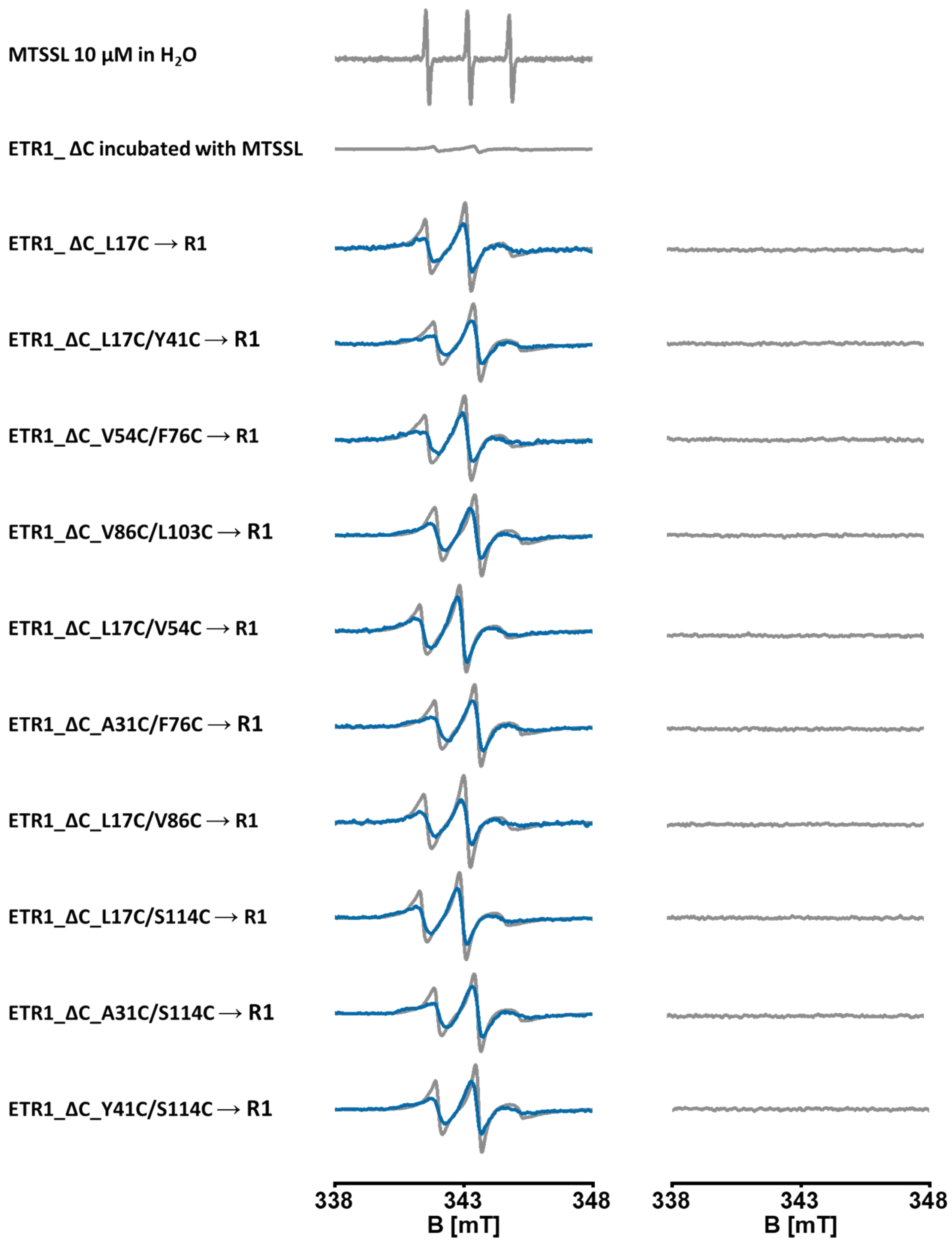

Room-temperature cw-EPR spectra of the unbound MTSSL or indicated ETR1 mutants. Left column: Spectra of the protein-detergent complex after spin labelling (grey), and after diamagnetic dilution and reconstitution (blue). The corresponding spectra on the left were normalized to the area, respectively. Right column: Spectra of the supernatant after ultracentrifugation (grey). No remaining nitroxide signal was found in the supernatant.

Figure S4: Non-reducing PAGE

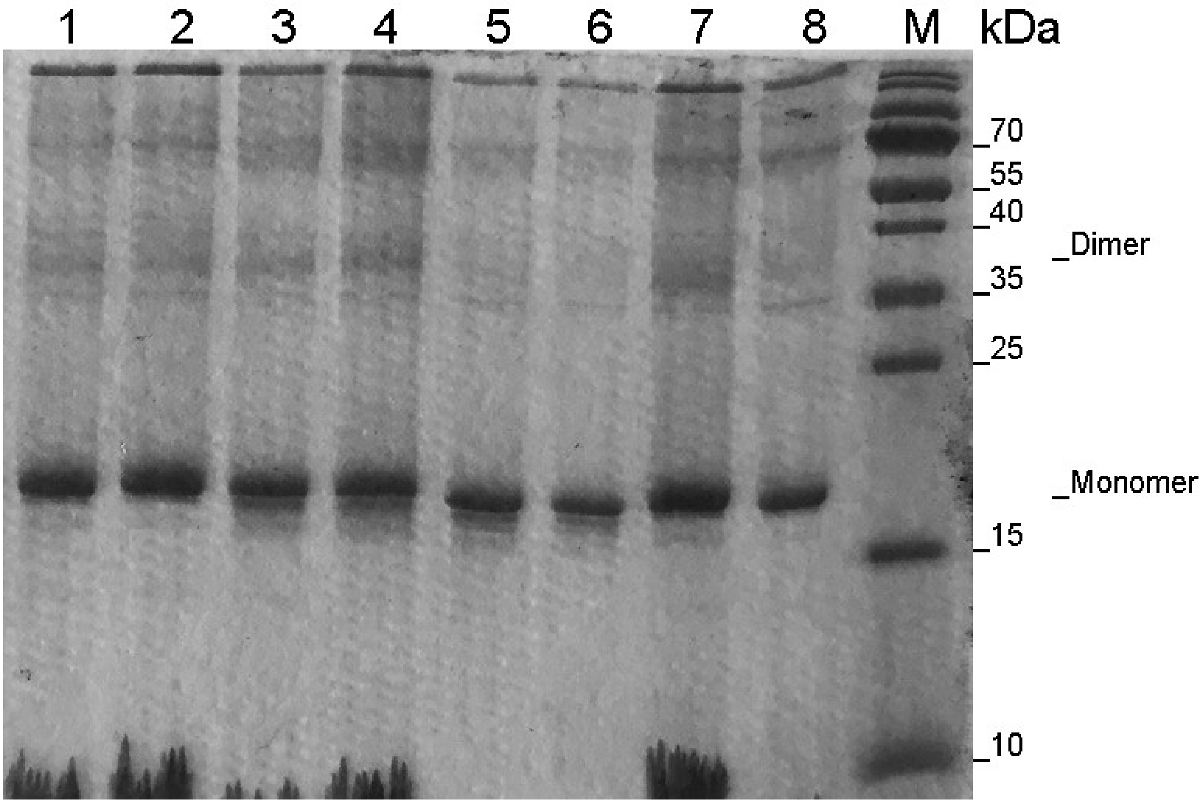

Non-reducing blue native PAGE of ETR1_TMD variants. Different combinations of spin labels (MTSSL and 3-maleimido-PROXYL) and reducing agents (TCEP and DTT) were probed for optimization of spin labelling. Lane 1-4: ETR1_ΔC_L17C, Lane 5-8: ETR1_ΔC_L17C/V54C. Lane 1 and 5: DTT + MTSSL, Lane 2 and 6: TCEP + PROXYL, Lane 3 and 7: DTT + PROXYL, Lane 54 and 8: TCEP + MTSSL. For all conditions, only insignificant dimer formation was found. Final spin labelling conditions were therefore optimized in terms of spin labelling degree. Proteins after preferred spin labelling conditions (use of DTT and MTSSL) are shown in lanes 1 and 5. Molecular weight markers for monomers and dimers are indicated.

Figure S5: DLS data of LUVs and proteoliposomes

A

Empty DMPC LUVS after extrusion

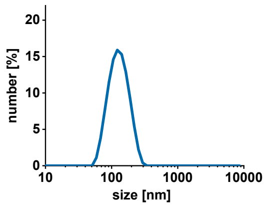

B

Proteoliposomes

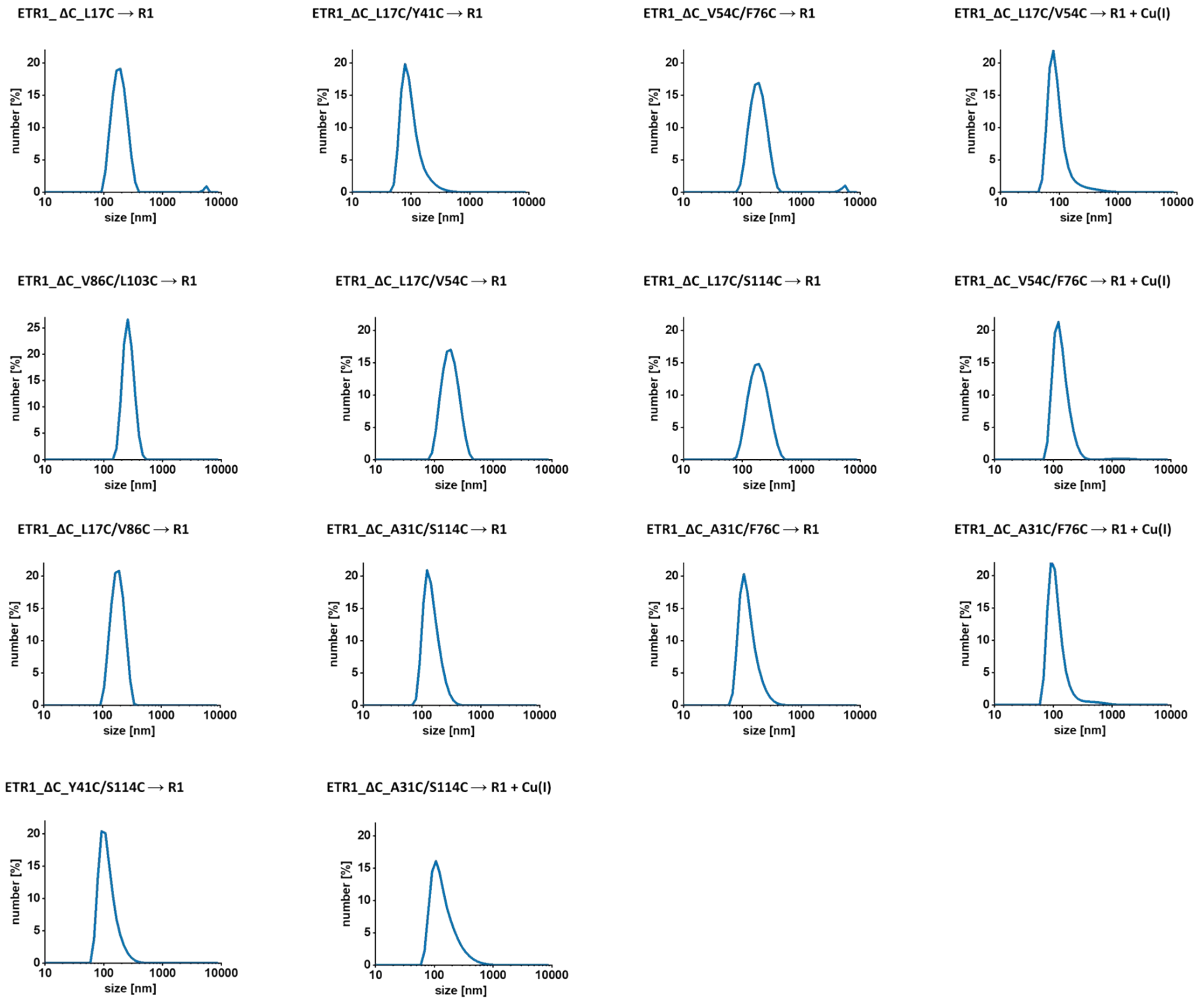

Dynamic light scattering (DLS) spectra of vesicles used in this study. The vesicle size of approx. 100 nm was confirmed by the DLS intensity. (A) DMPC vesicles extruded to 100 nm. (B) Proteoliposomes containing a mixture of spin-labelled ETR1 and ETR1_ ΔC for diamagnetic dilution.

Figure S6: DEER data of double mutants

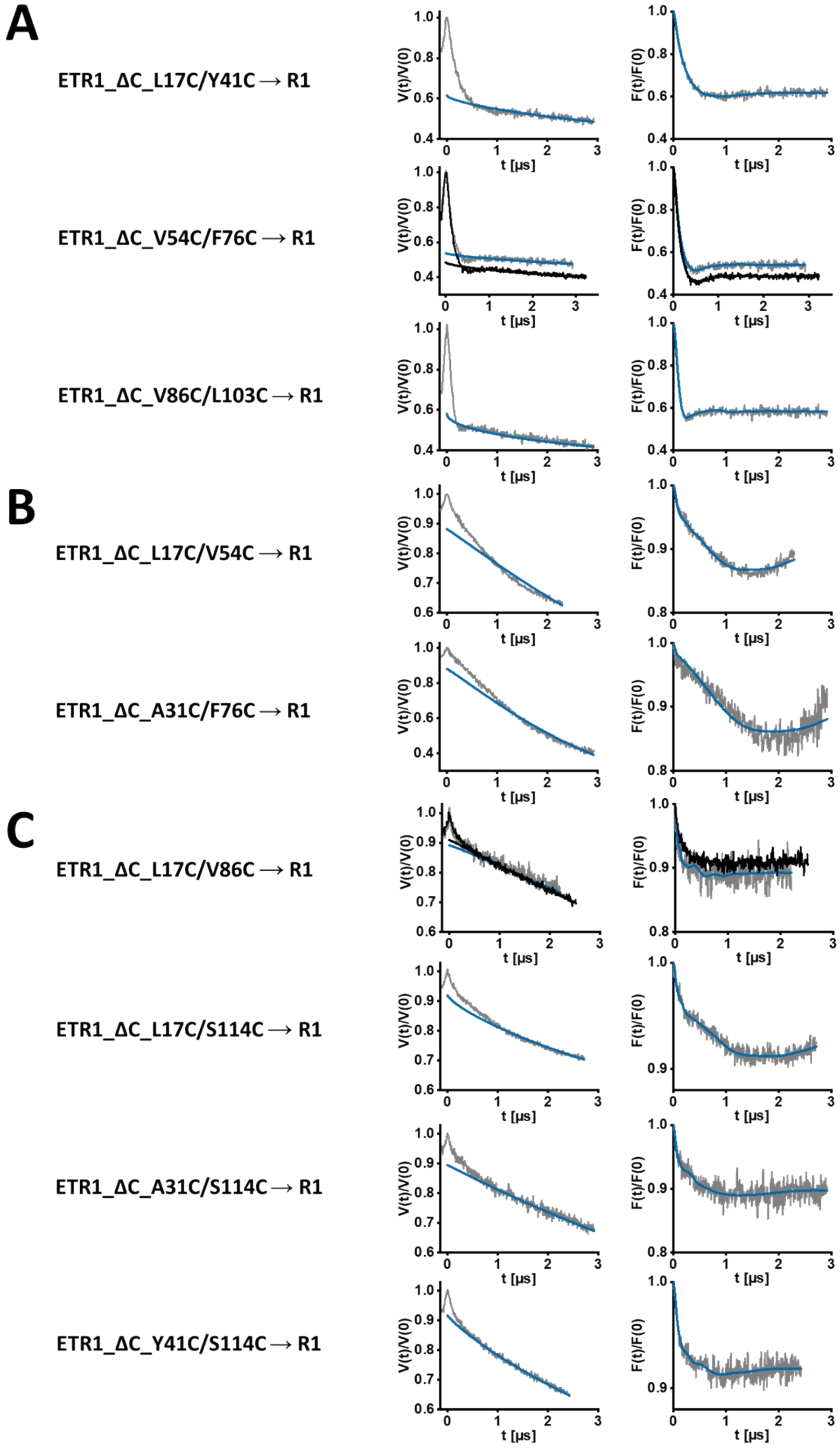

Evaluation of DEER measurements of the indicated doubly spin-labelled ETR1_TMD variants diamagnetically diluted and reconstituted. (A) intrahelical distances, (B) helix 1 ⟶ helix 2, (C) helix 1 ⟶ helix 3.Data were processed using MATLAB R2019b, the DeerAnalysis 2018a software[8], and DEERNet[10] 2019. Replicates of independently prepared samples are shown in black. Left column: Primary DEER signal (grey) and background fit (blue) as obtained by DEERNet. Right column: Background-corrected form factor (grey) and fit (blue) as obtained by DEERNet. Corresponding distance distributions for data from (A) and (C) are shown in Figure 3, main text.

Figure S7: Determination of copper-protein-stoichiometry

A

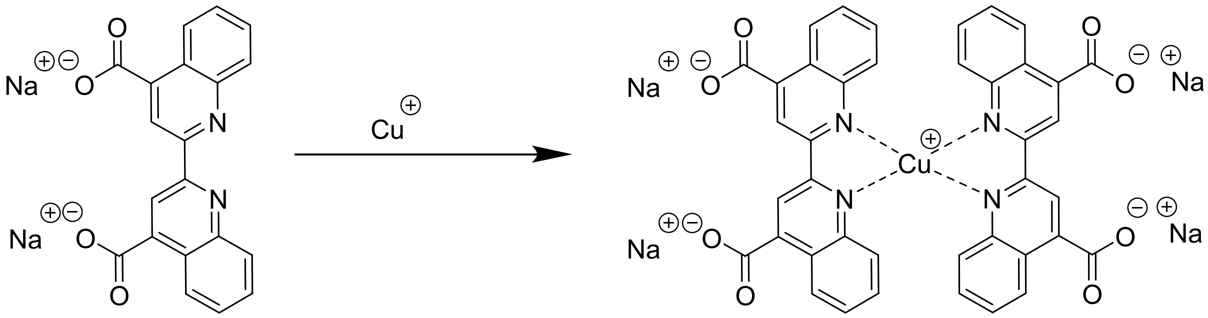

B

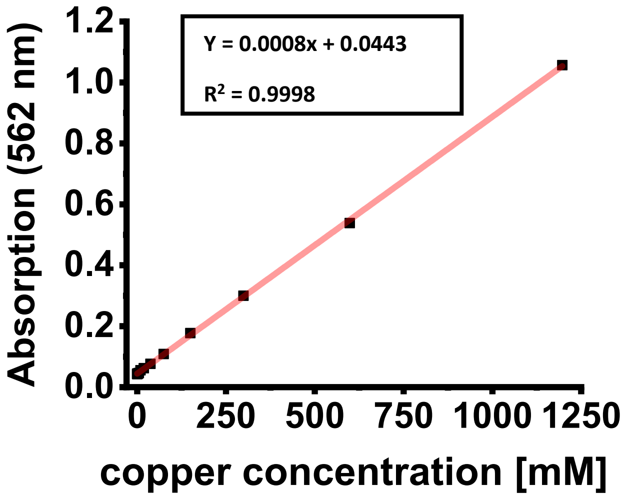

C

Mutant	Protein concentration [µM]	Cu(I) concentration [µM]	Cu(I)/protein ratio
**ETR1_ΔC_V54C/F76C → R1**	109	82	0.75
**ETR1_ΔC_L17C/V54C → R1**	521	197	0.38
**ETR1_ ΔC_A31C/F76C → R1**	105	102	0.97
**ETR1_ ΔC_A31C/S114C → R1**	146	128	0.88

A: Structural formula of Bicinchoninic acid (BCA). BCA forms a complex with Cu(I), which exhibits a strong absorption signal at 562 nm.[1]

B: Calibration curve of BCA_2-Cu(I).

C: Determination of copper-protein-stoichiometries of the indicated spin-labelled ETR1 variants.

Figure S8: DEER data of copper(I)-loaded variants

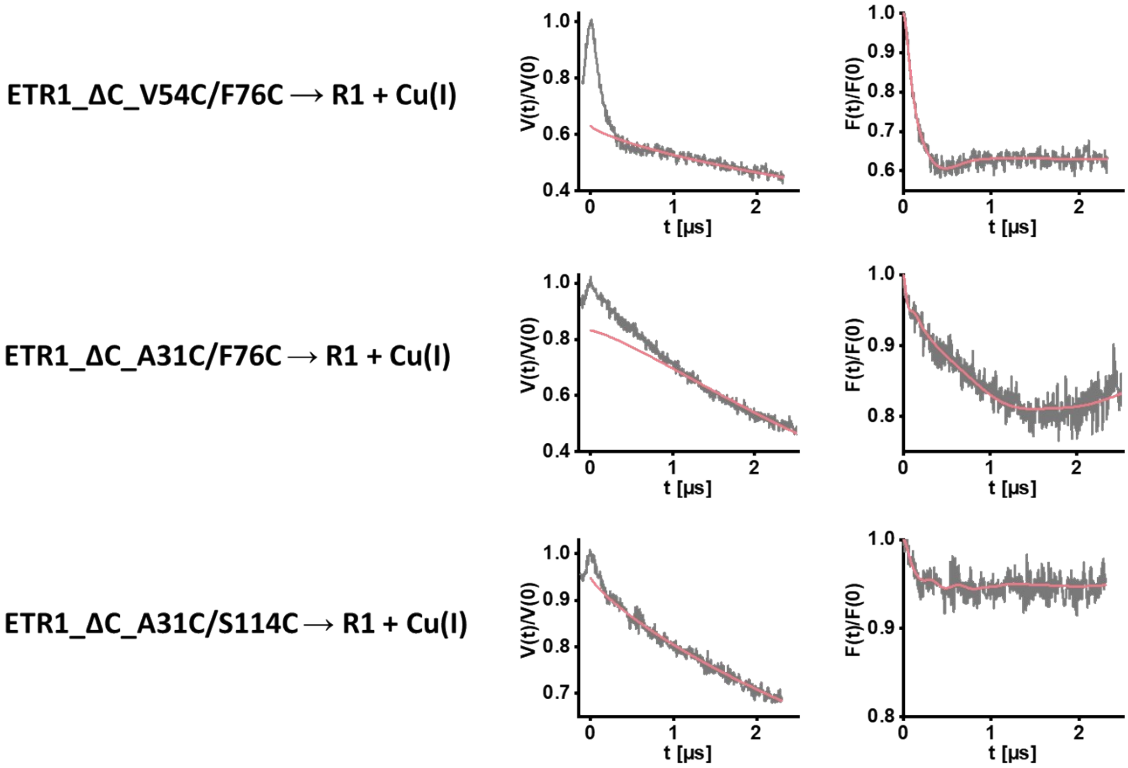

Evaluation of DEER measurements of the indicated doubly spin-labelled ETR1_TMD variants, loaded with Cu(I), diamagnetically diluted and reconstituted. Data were processed using MATLAB R2018a, the DeerAnalysis2019 software[8] and DEERNet[10]. Left column: Primary DEER signal (grey) and background fit (pink). Right column: Background-corrected form factor (grey) and fit (pink). Overlays of the form factors of samples with and without Cu(I) are shown in Figure 4C, main text.

Figure S9: Structural models used for distance simulations

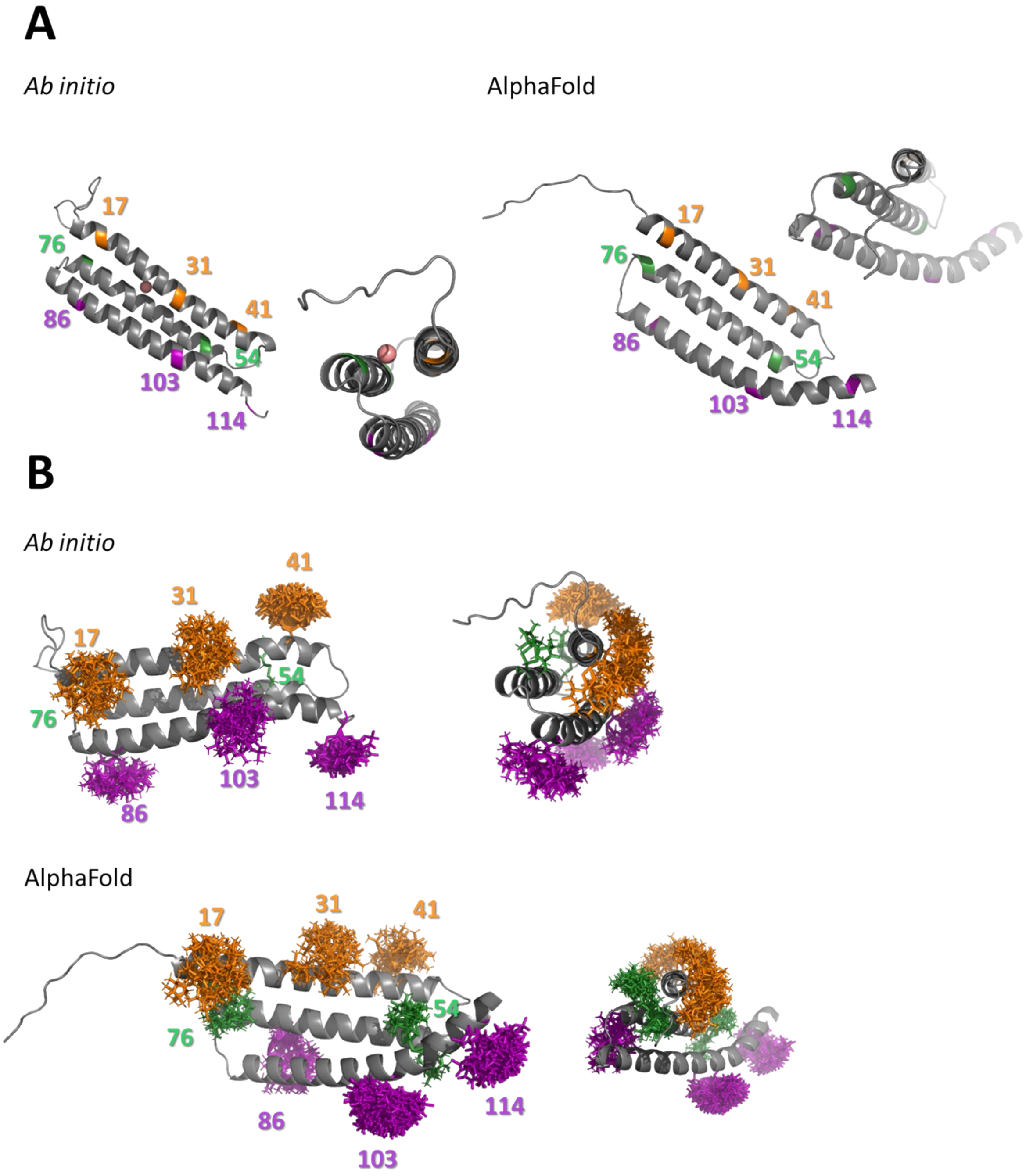

A: *Ab initio* predicted model[1] of the ETR1_TMD (left) and AlphaFold[14] structure (entry P49333, right). Side view onto the helix 1-helix 3 interface and top view onto helix 1 are shown, respectively. For the *ab initio* structure the position of the Cu(I) atom was included.

B: Possible MTSSL conformations (rotamers) attached to all labelling sites used in this study. The rotamers were calculated with the MATLAB program Multiscale Modeling of Macromolecules (MMM, version 2018.2).[15] Colour code: Helix 1 (orange), Helix 2 (green), Helix 3 (purple). Two different perspectives from the side (left) and top (right) are displayed. The resulting expected distance distributions are shown in Figure 3, main text.

Figure S10: Low-temperature cw-EPR spectra

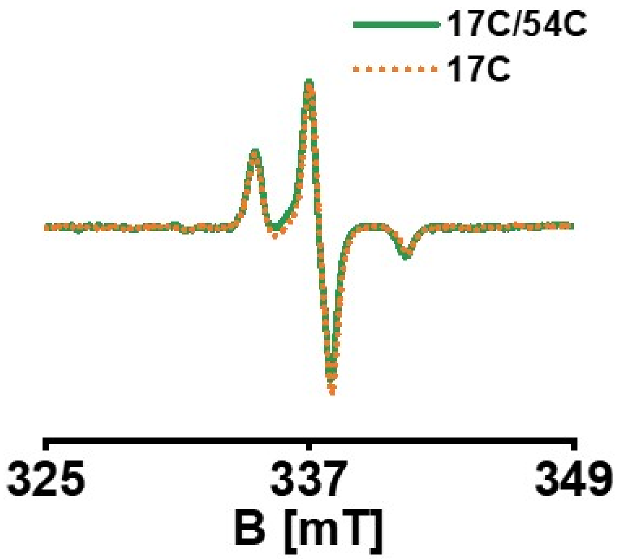

Cw-EPR spectra of the singly labelled variant (17C, orange dotted line) and the doubly labelled variant (17C/54C, green) were recorded at 120 K. After baseline-correction, the spectra were normalized to their integrated intensity. No deviations in peak amplitude were observed.

Figure S11: DEER data of single mutant

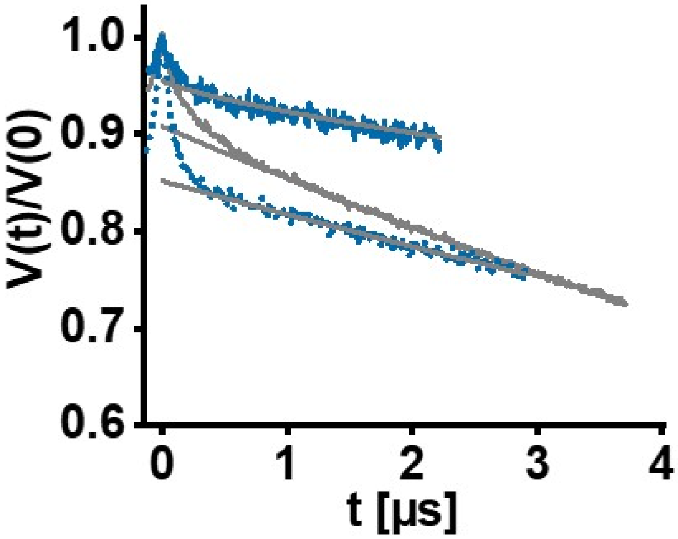

Primary DEER signal and background fit (grey) of ETR1_ΔC_L17C⟶R1 in detergent (grey line), after reconstitution (dashed blue line) and after diamagnetic dilution and reconstitution (solid blue line). Data were processed using MATLAB R2018a, the DeerAnalysis2019 software[8] and DEERNet[10]. The resulting form factors are shown in Figure 2C, main text.

Figure S12: CD spectra

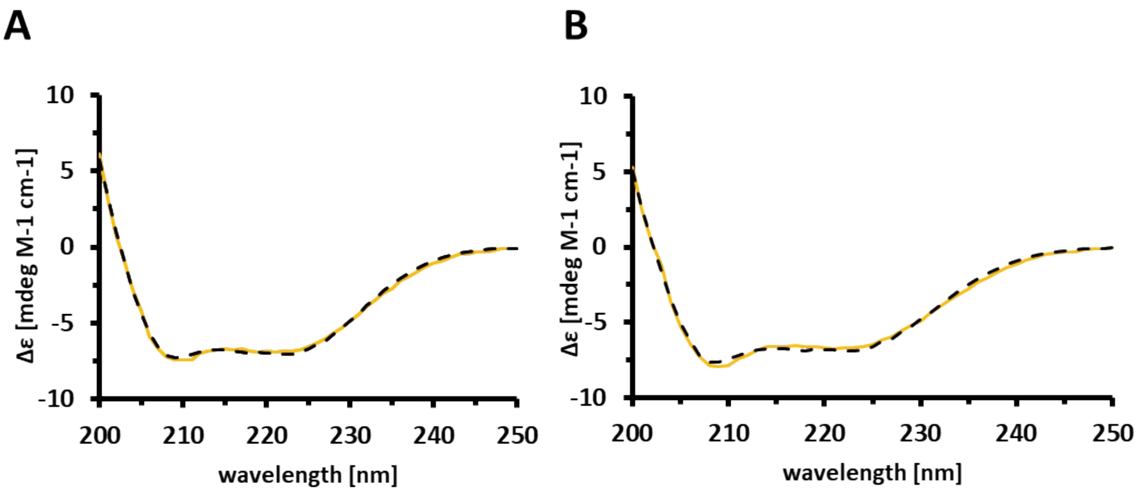

C

BESTSEL	α-Helix	β-Sheet	β-Turn	Random
**ETR1_TMD wild type**	66.4 %	0 %	11.6 %	22 %
**ETR1_TMD_ΔC**	63.7 %	0 %	8.4 %	27.9 %

Circular Dichroism (CD) spectra of purified ETR1_TMD wild type (A) and ETR1_TMD_ΔC (B). Spectra shown (orange lines) were recorded on a Jasco-715 spectropolarimeter (Jasco GmbH, Gross-Umstadt, Germany) at 0.1 nm resolution, 0.1 cm path length, 10 accumulations with a wavelength range from 250 to 200 nm. Secondary structures summarized in (C) were calculated from these spectra with BeSTSel.[11-13] Overall, the spectra of wild type and cysteine mutant are highly similar and correspond to previous CD data on receptor orthologs from Arabidopsis and tomato.[16, 17]

Supplementary References

1. S. Schott-Verdugo, L. Müller, E. Classen, H. Gohlke and G. Groth, *Sci. Rep.*, 2019, **9**, 1-14.
2. E. Bordignon, in *EPR Spectroscopy*, Springer, 2011, 121-157.
3. E. Gasteiger, C. Hoogland, A. Gattiker, M. R. Wilkins, R. D. Appel and A. Bairoch, *The proteomics protocols handbook*, 2005, 571-607.
4. J.-L. Rigaud and D. Lévy, in *Methods Enzymol.*, Elsevier, 2003, vol. 372, 65-86.
5. N. Le Breton, T. Adrianaivomananjaona, G. Gerbaud, E. Etienne, E. Bisetto, A. Dautant, B. Guigliarelli, F. Haraux, M. Martinho and V. Belle, *Biochim. Biophys. Acta, Bioenerg.*, 2016, **1857**, 89-97.
6. A. Scherer, S. Tischlik, S. Weickert, V. Wittmann and M. Drescher, *Magn. Reson.*, 2020, **1(1)**, 59-74.
7. C. E. Tait and S. Stoll, *Phys. Chem. Chem. Phys.*, 2016, **18**, 18470-18485.
8. G. Jeschke, V. Chechik, P. Ionita, A. Godt, H. Zimmermann, J. Banham, C. Timmel, D. Hilger and H. Jung, *Appl. Magn. Reson.*, 2006, **30**, 473-498.
9. O. Schiemann, C. A. Heubach, D. Abdullin, K. Ackermann, M. Azarkh, E. G. Bagryanskaya, M. Drescher, B. Endeward, J. H. Freed and L. Galazzo, *J. Am..Chem. Soc.*, 2021, **143**, 17875-17890.
10. S. G. Worswick, J. A. Spencer, G. Jeschke and I. Kuprov, *Sci. Adv.*, 2018, **4**, eaat5218.
11. A. Micsonai, F. Wien, É. Bulyáki, J. Kun, É. Moussong, Y.-H. Lee, Y. Goto, M. Réfrégiers and J. Kardos, *Nucleic Acids Res.*, 2018, **46**, W315-W322.
12. A. Micsonai, F. Wien, L. Kernya, Y.-H. Lee, Y. Goto, M. Réfrégiers and J. Kardos, *Proc. Natl. Acad. Sci.*, 2015, **112**, E3095-E3103.
13. A. Micsonai, É. Bulyáki and J. Kardos, in *Struct. Genomics*, Springer, 2021, 175-189.
14. J. Jumper, R. Evans, A. Pritzel, T. Green, M. Figurnov, O. Ronneberger, K. Tunyasuvunakool, R. Bates, A. Žídek and A. Potapenko, *Nature*, 2021, **596**, 583-589.
15. G. Jeschke, *Protein Sci.*, 2018, **27**, 76-85.
16. E. Classen and G. Groth, *Mol. Membr. Biol.*, 2012, **29**, 26-35.
17. M. Berleth, N. Berleth, A. Minges, S. Hänsch, R. C. Burkart, B. Stork, Y. Stahl, S. Weidtkamp-Peters, R. Simon and G. Groth, *Front. Plant Sci.*, 2019, **10**, 726.
